# Chondriokinesis during microsporogenesis in plants

**DOI:** 10.1007/s00425-017-2706-8

**Published:** 2017-05-08

**Authors:** Dorota Tchórzewska

**Affiliations:** Department of Plant Anatomy and Cytology, Maria Curie-Skłodowska University, Akademicka 19 Street, 20-033 Lublin, Poland

**Keywords:** Meiosis, Microsporogenesis, Cell organelles, Chondriokinesis

## Abstract

**Chondriokinesis represents a highly orchestrated process of organelle rearrangement in all dividing plant and animal cells, ensuring a proper course of karyokinesis and cytokinesis. This process plays a key role in male gametophyte formation.**

Chondriokinesis is a regular rearrangement of cell organelles, assuring their regular inheritance, during both mitotic and meiotic divisions in plant and animal cells. The universal occurrence of the process implies its high conservatism and its probable origin at an early stage of plant evolution. The role of chondriokinesis is not only limited to segregation of cell organelles into daughter cells, but also prevention of fusion of karyokinetic spindles and delineation of the cell division plane. Thus, chondriokinesis plays an indispensable role in mitosis and meiosis as one of the various factors in harmonised cell division, being a key process in the formation of viable cells. Therefore, disturbances in this process often result in development of abnormal daughter cells. This has far-reaching consequences for the meiotic division, as emergence of abnormal generative cells impedes sexual reproduction in plants. This review is focused on microsporogenesis, because various plants exhibit a problem with sexual reproduction caused by male sterility. In this paper for the first time in almost 100 years, it is presented a compilation of data on chondriokinesis proceeding during microsporogenesis in plants, and providing view of the role, mechanism, and classification of this process in male gametophyte formation.

## Introduction

In plants, a haploid generation of the male generative line, which is directly involved in sexual reproduction, emerges through a process called microsporogenesis in spermatophytes—gymnosperms and angiosperms. A crucial role in this complex multi-step process is played by meiotic division comprising karyokinesis (nuclear division) and cytokinesis (cytoplasm division). Karyokinesis consists of two stages; the first stage involves the reduction division of chromosomes and is characterised by recombination leading to exchange of genetic material between homologous chromosomes. This extremely important process results in increased genotypic diversity and adaptation to environmental fluctuations (Harrison et al. 2010; Wijnker and Schnitter 2013). The second stage of karyokinesis has a conservative, mitotic nature, finally producing four independent nuclei. The second step, cytokinesis, in a majority of monocotyledonous angiosperms, and some gymnosperms exhibits successive cytokinesis taking place during meiosis (Sheffield and Bell 1987; Brown and Lemmon 1988b; Furness and Rudall 1999). In this process, a callose wall is formed between two nuclei in the first karyokinesis stage, and the second stage proceeds within the dyad. In Bryophyta, Pteridophyta, and dicotyledonous angiosperms, simultaneous cytokinesis predominates—the cell wall is formed already at the end of meiosis after both stages of karyokinesis (Davis 1966; Kapil and Bhatnagar 1991; Shimamura et al. 2003; Brown et al. 2010; Brown and Lemmon 2013). Besides these two main types, intermediate cytokinesis types are distinguished (Murty 1964; Bhandari 1984; Blackmore and Crane 1998). Finally, four haploid cells with half the chromosome number of the mother cell are formed through meiosis.

As early as at the turn of the 19th and 20th centuries, it was observed that karyokinesis and cytokinesis were accompanied by characteristic rearrangements of cell organelles, a process called chondriokinesis, during cell division (Fullmer 1899). It was found later that cell organelles (chondrion) did not migrate in a random way during the cell division stage, but exhibited a specific pattern of cellular distribution (Marquette 1907, 1908; Michaelis 1955). In subsequent studies, the authors demonstrated, using a mathematical approach, that organelle partitioning is not precisely uniform, but is much more nearly uniform (Birky 1983; Birky and Skavaril 1984). Recent investigations have shown that movement and distribution of organelles proceed in a highly organised manner with the involvement of the cytoskeleton, which ensures high precision of the distribution (Sheahan et al. 2004; Tchórzewska et al. 2008; Tchórzewska and Bednara 2011). Already in 1924, Guilliermond was the first to discover that “changes in the chondriosome”, i.e. rearrangements of cell organelles, were as important as chromosome segregation and later, based on several observations, the first classification of chondriokinesis was systematised in 1938 (Bąkowski 1938). The proposed classification comprised four main types of chondriokinesis: neutral, capsular, polar, and equatorial. Additionally, intermediate types, e.g. capsular-polar chondriokinesis, and more complex types, e.g. neutral chondriokinesis equatorial during telophase have also been included. The key criterion for classification of the chondriokinesis types was the arrangement of cell organelles during two meiosis phases: metaphase I and telophase I. The first comprehensive description of numerous variants of chondriokinesis described by Bąkowski indicates a large variety of rearrangements of cell organelles during cell division, characterising different plant species and in some animals.

Chondriokinesis later on was recognized as a very important process, as it involves migration of semi-autonomous organelles, such as plastids and mitochondria. These organelles with their own DNA are involved in the so-called cytoplasmic inheritance; therefore, their precise distribution to daughter cells determines formation of identical, viable microspores (Chase 2006). Furthermore, disturbances in the distribution of these organelles often cause cytoplasmic male sterility (Holford et al. 1991; Majewska-Sawka and Sadoch 2003). However, it is currently thought that grouping and migration of cell organelles is vital not only for precise distribution thereof into daughter cells, but rearrangements of cell organelles ensure an efficient course of cell division. For instance, in meiosis with simultaneous cytokinesis, in which the cell wall is formed only at the end of the process, i.e. during telophase II, there are two rounds of chromosome separation within one cell and cell organelle groups as equatorial plates, spatially limiting karyokinesis sites. It is postulated that the presence of an equatorial organelle plate prevents fusion of separating chromosomes or emerging karyokinetic spindles (Kudlicka and Rodkiewicz 1990; Rodkiewicz et al. 1992; Bednara et al. 1986, 1995; Tchórzewska et al. 1996, 2008; Brownfield et al. 2015). Additionally, the course of meiosis also depends on formation of the successive configurations of the microtubular cytoskeleton and plastids play a crucial role in this process, which has been described in numerous analyses of the meiosis process in monoplastid plant species (Brown and Lemmon 1982a, 1985, 1987a, b, 1988a, 1990, 1991a, 2004). Moreover, the phenomenon of cell polarity, which is extremely important for cell and tissue differentiation, depends on various external and internal factors (Noher de Halac and Harte 1985). In terms of the internal factors, irrespective of tissue interactions and additional metabolic factors during meiosis, cell polarity is influenced by formation of vacuoles, migration of the nucleus, dispersion of starch, formation of callose, and, particularly relevant, distribution of organelles (Ekici and Dane 2004). It should also be mentioned that the significant role of cell organelles is not limited to meiocytes, as it has been reported that plastids, which are located in the different cell layers of the microsporangium, serve various very important functions contributing to formation of the functional male gametophyte (Nepi et al. 1996; Clement and Pacini 2001). Thus, it can be pointed out that plastids and mitochondria, apart from their canonical indispensable role in energy metabolism, were adopted to perform additional equally important functions facilitating cell division and differentiation.

This paper, for the first time in almost 100 years, provides a comprehensive overview of information on the chondriokinesis process exclusively during microsporogenesis in plant species. The first survey along with classification developed in 1938 was based on single studies performed at the end of the nineteenth and the beginning of the twentieth centuries. Those investigations were conducted with limited methods and research tools, which substantially reduced the insight into the chondriokinesis process. The significant technological progress achieved later not only allowed validation of the previous observations, but also, what is more important substantially expanded our knowledge about meiotic division in plant cells. Thus, it provides the first complete review on chondriokinesis in microsporogenesis, taking into account numerous plant species. Although the study is focused on microsporogenesis, given the abundant literature on the sporogenesis, in the manuscript I took into account chondriokinesis process in monoplastid species, described as a new type of chondriokinesis, which has never been classified before. Additionally, this report provides a current view on the function and mechanism of chondriokinesis, thereby significantly extending the knowledge of this very important process in male gametophyte formation.

## Types of chondriokinesis

### Chondriokinesis in early prophase meiocytes

As described for various plants, the sexual life cycle, i.e. the transformation of a diploid sporophyte generation into a haploid gametophyte, is a result of meiotic division, consisting of karyokinesis and cytokinesis as well as the no less important process of chondriokinesis. In the male generative line, meiotic division occurs in pollen mother cells (PMC). Rearrangement of cell organelles, sometimes extremely dynamic, takes place in PMC as early as during early prophase I. The process was observed at the beginning of the twentieth century and described as “cytoplasmic granularities” grouping during prophase I (Marquette 1907; Jungers 1934; Lenoir 1934); however, mitochondria were not distinguished from plastids at that time, hence the descriptions were not precise. Currently, meiosis with both successive and simultaneous cytokinesis is characterised by the presence of various arrangements of plastids and mitochondria in prophase cells. There can be one or two groups of plastids and mitochondria (Albertsten and Palmer 1979; Bednara et al. 1986; Rodkiewicz and Duda 1988; Rodkiewicz et al. 1988a, b; Giełwanowska et al. 2003); a group of plastids and some mitochondria, with other mitochondria surrounding the cell nucleus (Rodkiewicz et al. 1988b, c, d; Brown and Lemmon 2001a); a separate group of plastids and a separate group of mitochondria (Geneves 1967, 1971; Audran 1964); and one group of plastids and mitochondria and another group of numerous endoplasmic reticulum cisternae located on the opposite side of the nucleus (Bednara and Rodkiewicz 1988). These organelle groupings are transient; as described below, they disperse in the cytoplasm and migrate, usually at the end of prophase I, in a way characteristic for each chondriokinesis type.

### Equatorial chondriokinesis

**Fig. 1:**
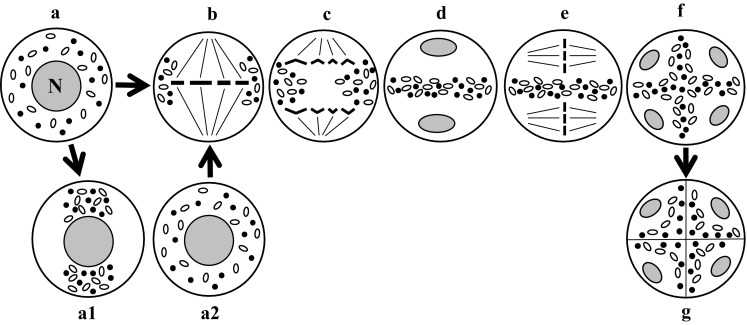
Equatorial chondriokinesis. *a* prophase I; *a1–a2* late prophase I in *Equisetum, Onoclea, Stangria,* and *Impatiens*; *b* metaphase I; *c* anaphase I; *d* telophase I; *e* metaphase II; *f* early telophase II; *g* late telophase II (Rodkiewicz and Duda 1988, amended). *Black spots* mitochondria; white spots plastids; *N* nucleus

Equatorial chondriokinesis is one of the four main types of the process (Fig. 1). Although plastids and mitochondria in this type are dispersed in the cytoplasm during prophase I (Fig. 1a), during metaphase I they group in the equatorial plane on both sides of the metaphase chromosome plate (Fig. 1b). Such an arrangement of organelles in the metaphase meiocyte classifies chondriokinesis to the equatorial type. Next, during anaphase I, the chondrion gradually disperses in the cytoplasm (Fig. 1c) and forms an equatorial plate between the daughter nuclei during telophase I (Fig. 1d). Cell organelles remain in this position during the second meiotic division (Fig. 1e) until telophase II and, after karyokinesis, they form other plate separating successive daughter nuclei (Fig. 1f). Within such an equatorial arrangement, the cell wall is formed at the end of telophase II (Fig. 1g). Equatorial chondriokinesis was described by Bąkowski (1938) only in meiosis occurring in animal sperm cells. The researcher claimed that this type of chondriokinesis did not take place in the world of plants. Subsequent investigations revealed that equatorial chondriokinesis was characteristic for many plant species. It was described in *Tradescantia virginica* (Rodkiewicz et al. 1984b, 1986), *Clarkia elegans* and *Lysimachia thyrsiflora* (Rodkiewicz et al. 1986), *Impatiens sultani* and *Lonicera japonica* (Brown and Lemmon 1988b), and *Cypripedium californicum* (Brown and Lemmon 1996, 1998). Furthermore, at the end of the twentieth century, numerous analyses focused on the distribution of plastids and mitochondria in plant cells provided a detailed description of the organisation of the chondrion in early prophase meiocytes. Cytological analyses of meiosis in *Equisetum hyemale* (Bednara and Giełwanowska (1987); Bednara and Rodkiewicz 1985; Bednara et al. 1986, 1995; Rodkiewicz et al. 1986, 1992), *E. fluviatile* (Lehmann et al. 1984; Bednara et al. 1986), *E. palustre* (Bednara and Giełwanowska (1987); Bednara et al. 1986), *E. variegatum* (Bednara et al. 1986), *Onoclea sensibilis* (Marengo 1977; Rodkiewicz and Duda 1988), *Stangeria eriopus* (Rodkiewicz et al. 1986, 1988a, 1992; Rodkiewicz and Duda 1988), and *Impatiens balsamina* (Dupuis 1978; Rodkiewicz et al. 1984b, 1986, 1988a, 1992) showed that plastids and mitochondria of these species formed two groups visible at the two cell poles during late prophase I (Fig. 1a1). Such groupings are visible transiently during prophase I, and the organelles disperse in the cytoplasm at the end of this phase (Fig. 1a2). In the successive stages, the organelle rearrangements correspond to equatorial chondriokinesis; therefore, although this process in *Equisetum, Onoclea*, *Stangria,* and *Impatiens* initially has a polar character, it can be regarded as equatorial chondriokinesis as the organelles are grouped in the equatorial plane during metaphase I.

### Neutral chondriokinesis equatorial during telophase

**Fig. 2:**
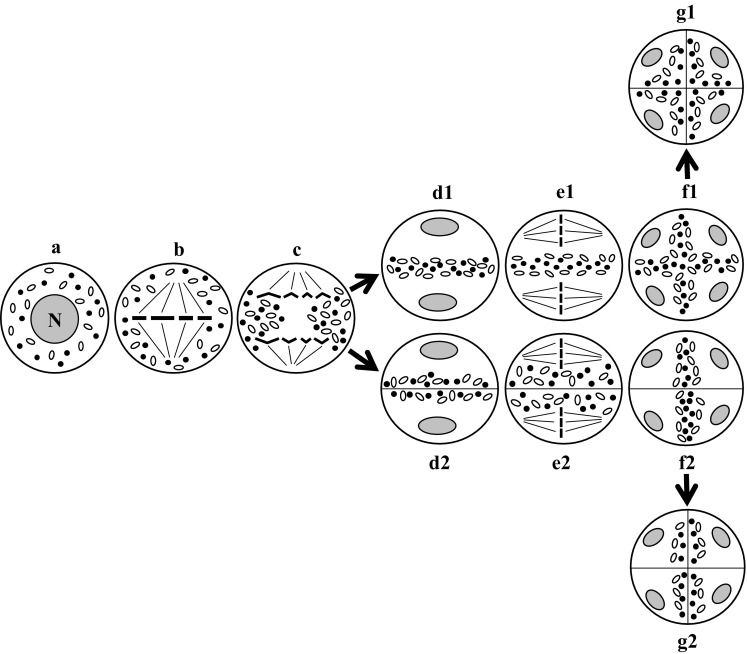
Neutral chondriokinesis equatorial during telophase: *a-g1* microsporogenesis with simultaneous cytokinesis, *a-g2* microsporogenesis with successive cytokinesis. *a* prophase I; *b* metaphase I; *c* anaphase I; *d1, d2* telophase I; *e1, e2* metaphase II; *f1, f2* early telophase II; *g1, g2* late telophase II (Bąkowski 1938, amended)

The most frequently described type is neutral chondriokinesis equatorial during telophase, which occurs in the meiosis stage with simultaneous cytokinesis (Fig. 2a–g1). In this complex chondriokinesis, cell organelles are uniformly distributed in the cytoplasm not only in prophase (Fig. 2a) but also in metaphase (Fig. 2b) meiocytes. They persist in this arrangement until anaphase I, during which they gradually move towards the equatorial plane of the cell (Fig. 2c). During telophase I, an organelle equatorial plate is formed between the daughter nuclei (Fig. 2d1) and the second meiotic division takes place within the meiocyte cytoplasm delineated by the plate composed of plastids and mitochondria (Fig. 2e1). Next, during anaphase II, the chondrion moves between the four forming daughter cells to separate them during telophase II (Fig. 2f1). At the end of telophase II, simultaneous cytokinesis takes place and the cell wall is formed within the organelle plates (Fig. 2g1). This type of chondriokinesis was described by Suessenguth (1921) in *Chamaedorea Karwinskiana* and Sugiur (1928) in *Tropaeolum peregrinum*. It was also included in the classification proposed by Bąkowski (1938). Neutral chondriokinesis equatorial during telophase was identified in the investigations of *Ribes rubrum* (Geneves 1967, 1971), *Podocarpus macrophylla* (Vasil and Aldrich 1970), *Paeonia tenuifolia* and *Campanula rapanculoides* (Dietrich 1973), *Pteridium aquilinum* (Sheffield and Bell 1979), *Dryopteris borreri* (Sheffield and Bell 1979; Sheffield et al. 1983), *Lycopersicon peruvianum* (Pacini and Juniper 1984), *Datura inoxa*, *Nicotiana tabacum* and *Antirrhinum majus* (Dupuis et al. 1988), *Solanum nigrum* (Bhandari and Sharma 1988), *Polystichum loncitis* (Bednara and Rodkiewicz 1988), “Sabine Queen” orchid (Brown and Lemmon 1991b), *Lilium longiflorum* (Dickinson and Heslop-Harrison 1970; Tanaka 1991), *Ophioglossum petiolatum* (Brown and Lemmon 2001a, b), *Psilotum nudum* (Lee 1982; Gabarayeva 1985; Tchórzewska et al. 1996; Tchórzewska and Bednara 2011), *Ginkgo biloba* (Wolniak 1976; Wang et al. 1988; Brown and Lemmon 2005), *Taranna gracilipes* (Vinckier and Smets 2007), *Armoracia rusticana* (Winiarczyk et al. 2007), and *Arabidopsis thaliana* (Brownfield et al. 2015).

Neutral chondriokinesis equatorial during telophase was also observed during meiosis with successive cytokinesis (Fig. 2a–g2) in such species as *Larix europea* (Rodkiewicz et al. 1984b; Bednara and Rodkiewicz 1988), “Vista Rainbow” orchid (Brown and Lemmon 1991b), and *Tinantia erecta* (own unpublished observations). The cell organelles in these plants behave during the first meiotic division in the same way as in the chondriokinesis type described above, i.e. in prophase and metaphase meiocytes, they are uniformly distributed until anaphase I and next they gradually move towards the equatorial plane (Fig. 2a–c) and form an equatorial organelle plate between daughter nuclei during telophase I. However, at the end of telophase I, a callose wall is formed in the organelle plate (Fig. 2d2), unlike during meiosis with simultaneous cytokinesis, and the second meiotic division proceeds in the bi-cellular meiocyte (Fig. 2e2). In this case, the cell organelles are still dispersed in the cytoplasm until late anaphase II. Then, they gradually move again towards the equatorial plane between the emerging daughter nuclei and form an organelle plate during telophase II (Fig. 2f2). This is followed by the second stage of successive cytokinesis and a cell wall between the daughter nuclei is formed within the organelle band (Fig. 2g2). In summary, it can be concluded that the cell wall formed after the first meiotic division delineates sites in the cell where karyokinesis takes place. Consequently, the cell organelles can be dispersed and evenly distributed in the meiocyte cytoplasm during the second meiotic division, which is not observed at simultaneous cytokinesis, and there is no wall after the first meiotic division.

### Lateral chondriokinesis equatorial during telophase

**Fig. 3:**
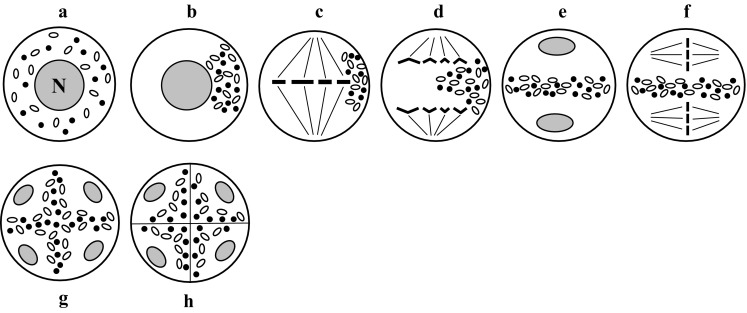
Lateral chondriokinesis equatorial during telophase. *a* early prophase I; *b* late prophase I; *c* metaphase I; *d* anaphase I; *e* telophase I; *f* metaphase II; *g* early telophase II; *h* late telophase II (Rodkiewicz and Duda 1988, amended)

Lateral chondriokinesis equatorial during telophase was described by Bąkowski in *Marsilia quadrifolia* based on the single study by Marquette (1908). Initially, cell organelles are dispersed in the cytoplasm in this type of chondriokinesis (Fig. 3a); next, they are grouped on one side of the nucleus in the equatorial plane during late prophase I (Fig. 3b). This arrangement persists during metaphase I, beside the metaphase chromosome plate (Fig. 3c); next, the group moves towards the equatorial plane of the cell during anaphase I (Fig. 3d). During the subsequent meiotic phases (Fig. 3e–h), cell organelles move in a way that is characteristic for equatorial chondriokinesis with simultaneous cytokinesis, i.e. they form an equatorial organelle plate during telophase I and II, which is a barrier delineating cytoplasmic spaces where karyokinesis proceeds. Lateral chondriokinesis equatorial during telophase was later described in *Delphinium elatum* (Bednara et al. 1995), *Nymphaea alba* (Rodkiewicz and Duda 1988; Rodkiewicz et al. 1988a, 1989, 1992; Bednara et al. 1995), and *Ophioglossum vulgatum* (Giełwanowska et al. 2003).

### Capsular chondriokinesis

**Fig. 4:**
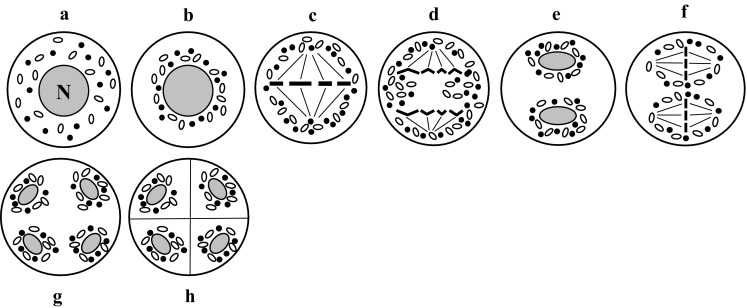
Capsular chondriokinesis. *a* early prophase I; *b* late prophase I; *c* metaphase I; *d* anaphase I; *e* telophase I; *f* metaphase II; *g* early telophase II; *h* late telophase II (Rodkiewicz and Duda 1988, amended)

Another main type is capsular chondriokinesis (Fig. 4), in which cell organelles are uniformly distributed in the cytoplasm at the beginning of prophase I (Fig. 4a), but at the end of this phase they are grouped in the form of a visible layer surrounding the cell nucleus (Fig. 4b). From this moment, karyokinesis in the first and second meiotic divisions proceeds in the cytoplasm delineated by a cell-organelle envelope. During metaphase I, organelles form a dense layer surrounding the karyokinetic spindle with metaphase chromosomes (Fig. 4c). During anaphase I, the organelles are temporarily dispersed (Fig. 4d) until formation of two daughter nuclei during telophase I; then they group again around the nuclei and form a dense layer (Fig. 4e). The second meiotic division occurs inside the capsules, which are mainly formed by plastids and mitochondria (Fig. 4f). Next, likewise during anaphase I, the organelles migrate during anaphase II and the cell organelle capsule is formed around the four nuclei emerging during telophase II (Fig. 4g). Simultaneous cytokinesis takes place at the end of telophase II (Fig. 4h).

Capsular chondriokinesis was initially described as perinuclear zones of dense granular cytoplasm; some researchers regarded it as aggregates of fixation artefacts (Luxenburg 1927). Nĕmec (1930) was the first to describe capsular chondriokinesis in *Larix decidua*. Next, this chondriokinesis type was described in *Lavatera* (Byxbee 1900), *Larix europea* (Devise 1922), *Gossypium* and *Althea* (Denham 1924), *Thespis* (Youngman 1927), and *Larix dahurica* (Prosina 1928). In the previous classification, Bąkowski (1938) cites only the reports of microsporogenesis observed in *Cucumis sativus* by Heimlich (1929) and the study performed by Migdalska (1934), who observed capsular chondriokinesis in *Gladiolus gandaviensis* and *G. primulinus*. This type of chondriokinesis was not verified in other species in further studies, with the exception of plants from the family Malvaceae (*Lavatera*, *Gossypium* and *Althea*). At present, capsular chondriokinesis is regarded as characteristic for all representatives of the family Malvaceae. To date, it has been described in *Malva sylvestris* (Rodkiewicz and Duda 1988; Rodkiewicz et al. 1988a; Kudlicka and Rodkiewicz 1990), *Lavatera trimestris* (Kudlicka and Rodkiewicz 1990), *Lavatera thuringiaca* (Tchórzewska et al. 2008, 2013), and *Gossypium arboreum* and *Alcea rosea* (Tchórzewska et al. 2013). Noteworthy is the finding reported recently, which shows that the characteristic grouping of organelles around the cell nucleus persists after the end of meiosis, even after the disintegration of the tetrad into single microspores found in *Gossypium arboreum* and *Lavatera thuringiaca* (Tchórzewska et al. 2013).

### Capsular chondriokinesis equatorial during telophase

**Fig. 5:**
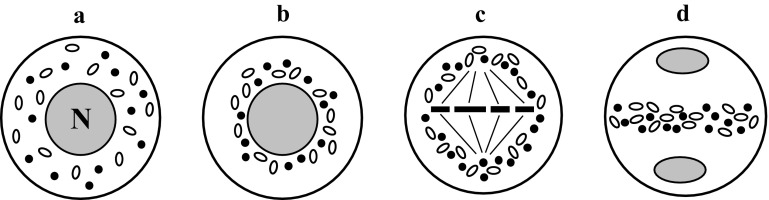
Capsular chondriokinesis equatorial during telophase. *a* early prophase I; *b* late prophase I; *c* metaphase I; *d* telophase I (Bąkowski 1938)

Capsular chondriokinesis equatorial during telophase represents the complex type of chondriokinesis. In this type of rearrangements, cell organelles are initially dispersed in the cytoplasm of prophase meiocytes (Fig. 5a), and form a capsule around the cell nucleus at the end of prophase I (Fig. 5b). As in the capsular type, the first meiotic division takes place within the space delineated by cell organelles (Fig. 5c). However, unlike in the capsular type, the organelles are dispersed during anaphase I and are grouped during telophase I as an equatorial plate between daughter nuclei (Fig. 5d). From this moment, chondriokinesis proceeds as the equatorial type. Such migrations of cell organelles during microsporogenesis have been observed in *Helleborus foetidus* (Nicolosi-Roncati 1910), *Ginkgo biloba* (Mann 1924), *Nephrodium molle* (Senjaninova 1927), *Equisetum palustre* (Lewitsky 1926), *E. limosum* (Jungers 1934), and *Hemerocallis fulva* fl. *pleno* (Fullmer 1899; Sienicka 1929), which was included in the classification proposed by Bąkowski (1938). Later, this type of chondriokinesis was confirmed in *Helleborus foetidus* (Echlin and Godwin 1968) and described in *Chondrilla juncea* (Kościńska-Pająk and Bednara 2003).

### Lateral capsular chondriokinesis equatorial during telophase

**Fig. 6:**
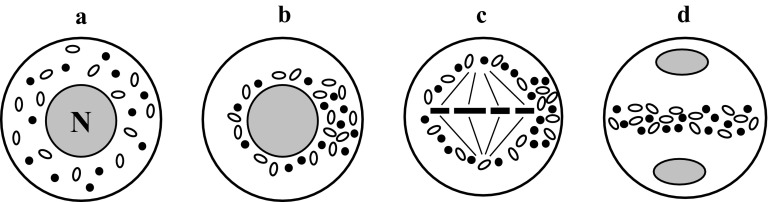
Lateral capsular chondriokinesis equatorial during telophase. *a* early prophase I; *b* late prophase I; *c* metaphase I; *d* telophase I; *e* prophase II (Bąkowski 1938, amended)

Lateral capsular chondriokinesis equatorial during telophase (Fig. 6a–d) is a variation of capsular chondriokinesis equatorial during telophase (described above), with the organelle capsule surrounding the dividing chromosomes on one side of the nucleus (Fig. 6c). Such grouping was described in *Petunia violacea* (Matsuda 1928) and *Nymphaea alba* (Guignard 1898) and included in the classification developed by Bąkowski (1938). However, subsequent investigations did not confirm the lateral capsular chondriokinesis equatorial during telophase in *Nymphaea alba*, which exhibited lateral chondriokinesis equatorial during telophase (Rodkiewicz and Duda 1988; Rodkiewicz et al. 1988a, 1989, 1992; Bednara et al. 1995).

### Neutral chondriokinesis

**Fig. 7:**
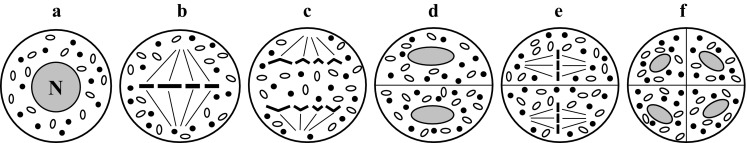
Neutral chondriokinesis. *a* prophase I; *b* metaphase I; *c* anaphase I; *d* telophase I; *e* metaphase II; *f* telophase II (Bąkowski 1938, amended)

The neutral chondriokinesis type was first described in the meiocytes of *Tetraclinis articulata* (Saxtona 1913) and *Equisetum variegatum* (Lenoir 1934), and this was mentioned in the classification by Bąkowski (1938). The species are characterised by meiosis with successive cytokinesis proceeding during microsporogenesis, i.e. the second meiotic division takes place in the dyad. In this type of chondriokinesis, cell organelles are uniformly dispersed in the meiocyte cytoplasm throughout the meiosis stages (Fig. 7a–f). In recent years, this type has been identified in *Allium sativum* (Winiarczyk 2009) and *A. ampeloprasum* (own unpublished observations).

### Neutral-polar chondriokinesis

**Fig. 8:**
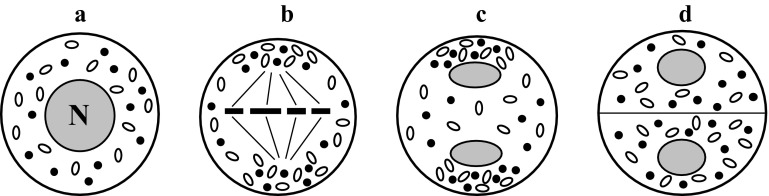
Neutral-polar chondriokinesis. *a* prophase I; *b* metaphase I; *c* telophase I; *d* prophase II (Bąkowski 1938)

In neutral-polar chondriokinesis, cell organelles are uniformly distributed in the cytoplasm in all the meiosis stages (Fig. 8a–d). However, their concentration is higher at the cell poles during metaphase I (Fig. 8b) and telophase I (Fig. 8c). This chondriokinesis type was observed in *Lupinus albus* (Milovidov 1928) and *Equisetum limosum* (Becker and Siemaszko 1936) and was included in the classification proposed by Bąkowski; however, to date, these data have not been verified and no reports of this type of chondriokinesis in meiosis have been published.

### Polar chondriokinesis

**Fig. 9:**
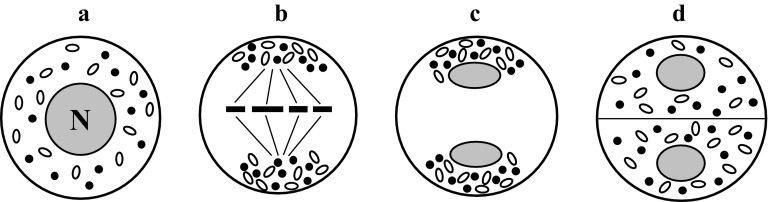
Polar chondriokinesis. *a* prophase I; *b* metaphase I; *c* telophase I; *d* prophase II (Bąkowski 1938)

According to the classification developed by Bąkowski, polar chondriokinesis is one of the four main types of the process. In this type, cell organelles are dispersed in the cytoplasm during prophase I (Fig. 9a) and accumulated at the opposite poles of the cell during metaphase I (Fig. 9b). They persist in these polar clusters until telophase I (Fig. 9c), i.e. until formation of the cell wall and a dyad. Plastids and mitochondria in the bi-cellular meiocyte are uniformly distributed in the cytoplasm (Fig. 9d) and such an arrangement persist until the end of meiosis. This type of chondriokinesis during meiosis was only described by Gugnard (1898) in *Limodorum abortivum*. It should be emphasised that although Bąkowski classifies polar chondriokinesis as the main type, there have been no other reports of polar chondriokinesis in microsporogenesis.

### Capsular-polar chondriokinesis

**Fig. 10:**
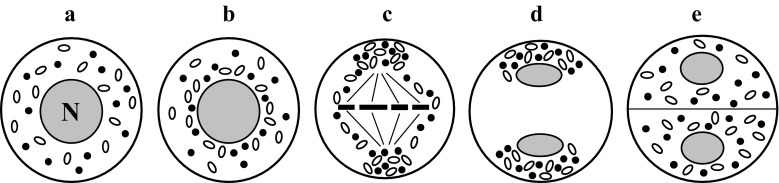
Capsular-polar chondriokinesis. *a* early prophase I; *b* late prophase I; *c* metaphase I; *d* telophase I; *e* prophase II (Bąkowski 1938, amended)

Capsular-polar chondriokinesis represents the complex type of the process, in which dispersed cell organelles in early prophase meiocytes (Fig. 10a) begin to group around the nucleus during late prophase (Fig. 10b). The first meiotic division proceeds within the characteristic capsule formed by the organelles, as in the capsular chondriokinesis type. However, a majority of cell organelles in this arrangement are located at the cell poles (Fig. 10c). Next, during telophase II, all organelles are arranged at the two cell poles, as in polar chondriokinesis (Fig. 10d). After formation of the callose wall between the daughter nuclei during the second meiotic division, cell organelles are uniformly distributed in the cytoplasm (Fig. 10e). Capsular-polar chondriokinesis was classified by Bąkowski (1938) based on the work on *Magnolia Yulan* published by Guignard (1898) and on the findings concerning meiosis in *Riccia Frostii* described by Black (1913). Later publications did not report this type of chondriokinesis.

### Monoplastidic chondriokinesis

**Fig. 11:**
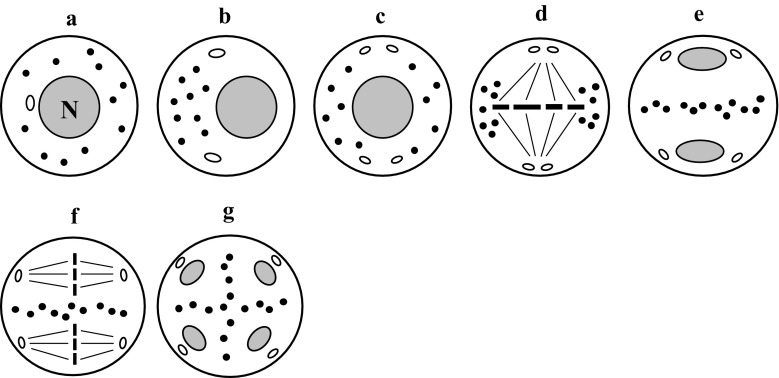
Monoplastidic chondriokinesis. *a* prophase I (early); *b* prophase I (leptoten-zygoten); *c* prophase I (pachyten-diploten); *d* metaphase I, *e* telophase I; *f* metaphase II; *g* telophase II (Brown and Lemmon 1988a, amended)

Unique plastid polarity during meiosis was observed in hornworts by von Mohl (1839), Nageli (1844), Strasburger (1880), and Davis (1899), but was never considered in Bąkowski’s classifications. This process was later studied using electron microscopy and immunofluorescence methods, providing more details of chondriokinesis in monoplastid liverwort, moss, and clubmoss species. In these species, early prophase sporocytes contain a single elongated plastid, a central nucleus, and mitochondria that are unevenly dispersed in the cytoplasm (Fig. 11a). In the later prophase, the nucleus moves towards an acentric position close to the plasma membrane, the plastid divides into two, and the mitochondria are still dispersed in the cytoplasm (Fig. 11b). At the end of prophase I, the two plastids divide again and four plastids are formed that are equidistantly positioned in the sporocyte cytoplasm in a tetrahedral arrangement. The nucleus migrates in this phase and is located centrally in the sporocyte at the end of prophase I (Fig. 11c). In the subsequent phase of meiosis, i.e. metaphase I, plastids are located at the cell poles close to the cytoplasmic membrane; in turn, mitochondria are grouped at the chromosomes in the cell equatorial plane in a manner typical of equatorial chondriokinesis (Fig. 11d). In consecutive meiosis phases, the mitochondria are translocated and grouped as in equatorial chondriokinesis, forming an equatorial plate during telophase I and II, which delineates spaces where karyokinesis takes place (Fig. 11e–g). In turn, plastids in monoplastid species exhibit a completely different arrangement than that of the mitochondria. Initially, they are located near the nuclear envelope (prophase I); in the successive phases of meiosis, they are arranged near the cell poles, often close to the cytoplasmic membrane (Fig. 11b–g). According to the criteria used by Bąkowski (1938), chondriokinesis in monoplastid species resembles the equatorial type due to the specific location of mitochondria in meiosis phases that are critical for classification (metaphase I and telophase I). In contrast, the polar arrangement of four plastids, which are associated with formation of karyokinetic spindles, indicates polar chondriokinesis in respect to rearrangement of plastids. Meiosis in monoplastid species was extensively described by Brown and Lemmon for *Rhynchostegium serrulatum* (Brown and Lemmon 1982a, b), *Amblystegium riparium* (Brown and Lemmon 1982c), *Atrichum undulatum* and *Entodon seductrix* (Brown and Lemmon 1987a, 1987b), *Marattia* (Brown and Lemmon 1997), *Angiopteris evecta* (Brown and Lemmon 2001a), *Phaeoceros laevis* and *Notothylas breutelii* (Brown and Lemmon 1990), *Isoetes melanopoda* (Brown and Lemmon 1987a, b, 1991a), and *Selaginella arenicola* (Brown and Lemmon 1985, 1991a).

## Role and mechanism of chondriokinesis

Chondriokinesis during meiosis is a widespread phenomenon in all analysed plant species, even from systematically distant groups (horsetails, mosses, ferns, Gymnosperms, and Angiosperms). The universal occurrence of the process implies its high conservatism and its probable origin at an early stage of plant evolution; however, the course of the process varies between plant species. It should be emphasised, that the role of chondriokinesis has not been synthetically presented as yet, and currently there are many data indicating varied relevance of the process in the meiotic division of the plant cell.

It is thought that the most fundamental role of the specific grouping of organelles throughout the phases of meiosis is to ensure equal distribution of organelles between the tetrad cells (Senjaninova 1927; Geneves 1967; Wolniak 1976; Dupuis 1978; Brown and Lemmon 1982b), which guarantees formation of identical, metabolically active microspores. This is confirmed by the fact that organelles, which are initially dispersed in the meiocyte cytoplasm, are grouped (e.g. neutral chondriokinesis equatorial during telophase) during the key meiosis phases (telophase I and telophase II), which ensures appropriate segregation into daughter cells. With time, when many biological phenomena were better explored at the molecular level, i.e. the importance of cytoplasmic inheritance and the role of semi-autonomous cell organelles in the formation of fertile pollen grains, chondriokinesis was assumed to ensure proper cytoplasmic inheritance of genetic material in the plant cell, wherein both the plastid and the mitochondrial genomes are equally responsible for cytoplasmic inheritance (Sears 1980; Hagemann and Schröder 1989; Kuroiwa 1991; Mogensen 1996; Nagata 2010). The impact of plastid or mitochondrial DNA on offspring traits has been described, especially restriction fragment length polymorphisms (RFLPs) were used to follow the organellar DNA inheritance (Boblenz et al. 1990; Derepas and Dulieu 1992; Hu et al. 1996; Trusty et al. 2007; Hansen et al. 2007; Matsushima et al. 2008b). In many plant species, the presence of plastid or mitochondrial DNA in male reproductive cells determining the potential for cytoplasmic inheritance has been shown (Zhang et al. 2003). Yet, this question seems debatable, as some authors claim that the presence of plastids in sperm cells does not indicate their inclusion in the zygote (Lombardo and Gerola 1968; Reboud and Zeyl 1994). It is, however, indisputable that cytoplasmic male sterility (CMS) is determined by a lack of the mitochondrial genome (Holford et al. 1991; Chase 2006; Wang et al. 2006). Hence, proper segregation of cell organelles is a key process in the formation of a fertile male gametophyte.

Besides the basic function of chondriokinesis (i.e. equal distribution of organelles and involvement in cytoplasmic inheritance), it was proposed that cell organelles constitute a barrier limiting the sites in the meiocyte cytoplasm in which karyokinesis takes place (Kudlicka and Rodkiewicz 1990; Rodkiewicz et al. 1992; Bednara et al. 1986, 1995; Tchórzewska et al. 1996, 2008; Brownfield et al. 2015). Organelles forming a capsule or an equatorial plate prevent fusion of karyokinetic spindles or separating chromosomes during the second meiotic division, serving as a “substitute of the cell plate” (Bednara et al. 1986). This idea was supported by the fact, that cell organelles were dispersed in the meiocyte cytoplasm even if they were grouped as an equatorial plate at the end of telophase I. This phenomenon has been described in many species characterised by meiosis with successive cytokinesis, in which neutral chondriokinesis equatorial during telophase occurs (Rodkiewicz et al. 1984b; Bednara and Rodkiewicz 1988; Brown and Lemmon 1991b). Additionally, in meiosis with successive cytokinesis, during neutral chondriokinesis equatorial during telophase, organelles are arranged in an equatorial plate during telophase II when another cell plate is formed. In this case, another function of chondriokinesis can be inferred, i.e. cell organelles are involved in cell plate formation (Rodkiewicz et al. 1986, 1988a, 1989).

Another function that can be assigned to chondriokinesis is related to the role of cell organelles in determination of meiocyte polarisation. This idea was formulated based on the observation of pollen development in gymnosperms, where plastids in the microspore tetrad remain close to the proximal wall of the tetrad after meiosis. The polarisation is important, as the prothallus cells develop at the proximal wall of pollen cells in gymnosperms and the pollen tube grows from the distal wall (Rodkiewicz et al. 1984a). The relevance of organelle grouping for polarisation of megasporocytes during megasporogenesis is particularly evident. Polarisation of meiocytes is extremely important in the process of female gametophyte development, given the competition between megaspores for formation of a functional megaspore, which will develop into the embryo sac (Steward and Gifford 1967; De Boer-de Jeu 1978; Willemse and Bednara 1979; Willemse and De Boer-de Jeu 1981; Bednara et al. 1981; Ekici and Dane 2004).

An additional function of chondriokinesis is the relationship between plastids and the organisation of tubulin cytoskeleton in the plant cell. This phenomenon has been described in numerous reports on meiosis in monoplastid plant species (Brown and Lemmon 1982a, 1985, 1987a, b, 1988a, 1991a, b, 2004, 2006). The acentrosomal spindle formed in plants begins polymerisation with γ-tubulin, which is the major component of microtubule organizing centres (MTOCs). Observations of meiosis in monoplastid plants revealed that γ-tubulin was located at the plastid envelope (Shimamura et al. 2004); therefore, the spindle apparatus is organised in association with plastid migration and division (Shimamura et al. 2003). Furthermore, division polarity has been described in Bryophyte meiosis, indicating that the prophase system of axially aligned microtubules determines the site of cytokinesis. Since microtubules are associated with plastids, the location of plastids determines the cell division plane (Brown and Lemmon 1987a).

The molecular mechanism of cell organelle transport and movement during cell division remains obscure. One of the first reports of the mechanism of organelle movement during meiosis suggests that this process is functionally linked to the cytoskeleton (Wolniak 1976). It should be emphasised that the phenomenon of migration of cellular organelles associated with the cytoskeleton has been repeatedly shown in animal cells (Stebbings 1990), lower plant cells (Menzel 1985; Busby and Gunning 1988), and higher plant cells (Tanaka 1991; Brownfield et al. 2015). Involvement of both the tubulin (MT) and actin (MF) cytoskeleton in migration of organelles was reported. Although most reports are primarily focused on somatic cells (Williamson 1993; Ligrone and Duckett 1998; Olyslaegers and Verbelen 1998; Kandasamy and Meagher 1999), it can be assumed that, due to the prevalence of this phenomenon, the mechanism involved in cytoskeleton-assisted organelle movement is universal and can be referred to meiotically dividing generative cells. As shown in *Nicotiana tabacum* protoplast cells, during initial steps of cell division, organelles are surrounded by “actin baskets” and these baskets facilitate their preparatory organisation during cell division; subsequently the “actin baskets” lose their integrity and individual organelles are tethered to acting filaments, which form a dense cytoplasmic network (Kandasamy and Meagher 1999; Sheahan et al. 2004). This leads to enmeshment of organelles and dependence of their location on dynamically changing cytoskeleton configurations (Sheahan et al. 2004). Consequently, organelles embedded at an appropriate place and time within cytoskeleton become considerably less mobile (Tirlapur and Konig 2001), which probably facilitates nearly equal distribution of cell organelles during cell division. Such a mechanism, with the involvement of the actin cytoskeleton, probably manages chondriokinesis in meiotically dividing cells, as indicated in *Psilotum nudum*. In this species, the destruction of the actin cytoskeleton during the consecutive stages of meiosis induced disturbances in the course of chondriokinesis (Tchórzewska et al. 2011). Some authors claim that organelle repositioning depends on actin filaments, but not on microtubules (Sheahan et al. 2004), however, analyses of the tubulin cytoskeleton in combination with capsular chondriokinesis in microsporogenesis in *Lavatera thuringiaca* showed a close relationship between MT configurations and the position of cell organelles. It seems that the radially oriented MTs around the nucleus in prophase I meiocytes (late leptotene), observed in *L. thuringiaca*, are associated with organelle assembly around the nucleus, which leads to formation of the species-specific organelle capsule. Later, tangentially oriented MTs in prophase I (diakinesis), which surround organelle clusters around the forming chromosomes, were described in this species. These observations indicate that, by limiting the space in the cell, the characteristic MT configuration maintains cell organelles in a strictly specified space around the forming chromosomes, which probably leads to reduced dynamics of organelle movement and thus allowing their nearly equal distribution. This conclusion is confirmed by investigations showing that the destruction of the tubulin cytoskeleton not only caused disturbances in the course of karyokinesis but also affected the chondriokinesis pattern (Tchórzewska et al. 2008).

In summary, it can be concluded that MTs and MFs play an active role in organelle redistribution within the cell, but depending on the type of cell and the phase of its life cycle, the movement mechanism is based on the tubulin and/or actin cytoskeleton.

## Conclusion

An important process in the complex generative reproduction of plants is the meiotic cell division, which ensures appropriate distribution of genetic material between daughter cells. The success of this process is determined by a number of orchestrated phenomena, with the prominent example of chondriokinesis, ensuring a proper course of karyokinesis and cytokinesis. It can be assumed that in the early evolution of plants, chondriokinesis simply determined efficient “nearly uniform” segregation of cell organelles together with their DNA, as can be proposed by mathematical modelling for simple systems, ensuring proper inheritance thereof, which has a fundamental role in the formation of daughter cells. However, it is thought that during the evolution of plant cells, chondriokinesis has acquired new functions related to support of karyokinesis, polarisation of meiocytes, determination of the division plane, and formation of the tubulin cytoskeleton. A hypothesis can, therefore, be proposed that the new functions of chondriokinesis acquired during plant evolution, which supports meiotic cell division, facilitated rapid and efficient division of generative cells with minimal energy costs. Thus, the emergence of efficient formation of the male gametophyte allowed reproductive success and dynamic plant adaptation to variable environmental conditions. The great number of types of organelle rearrangements taking place during meiotic division implies a high diversity of the process in the evolution, which is characteristic and constant not only within a species but also within large plant groups, e.g. a family (*Malvaceae*). The molecular mechanism of chondriokinesis has not been fully explored; however, it can be claimed that the cell organelle movement is driven by the tubulin and/or actin cytoskeleton.

**Fig. 12:**
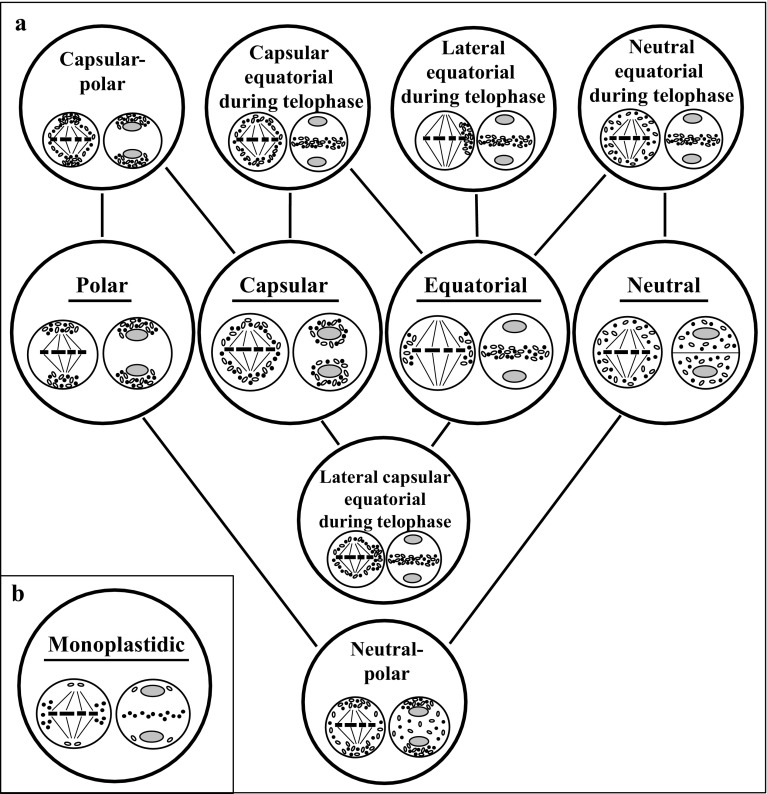
Graphic summary of all chondriokinesis types identified in sporogenesis or microsporogenesis. *A* classification developed by Bąkowski (1938). *B* unclassified chondriokinesis in monoplastid species

This paper provides the first complete classification of chondriokinesis. Importantly, the role of chondriokinesis was comprehensively illustrated, showing its indispensable role in meiosis, as one of the various factors in harmonised cell division. Graphic representation of all the chondriokinesis types described above is provided in Fig. 12. The types of chondriokinesis taking place during meiosis with the names of species and authors are shown in Table 1. The table presents species (marked in bold) that exhibit different types of organelle arrangement than that shown by Bąkowski in his classification (1938), as indicated in later studies. Furthermore, the table shows monoplastid chondriokinesis, which has not been classified so far.

**Table 1:** Chondriokinesis types identified in sporogenesis or microsporogenesis in plant species

Chondriokinesis		Species	Author
M	Equatorial	*Onoclea sensibilis*	Marengo (1977); Rodkiewicz and Duda (1988)
		*Impatiens balsamina*	Dupuis (1978); Rodkiewicz et al. (1984a, b, 1986, 1988a, 1992)
		*Tradescantia virginica*	Rodkiewicz et al. (1984a, b, 1986)
		*Equisetum variegatum*	Bednara et al. (1986)
		*Equisetum palustre*	Bednara and Giełwanowska (1987), Bednara et al. (1986)
		*Equisetum fluviatile*	Lehmann et al. (1984); Bednara et al. (1986)
		*Equisetum hyemale*	Bednara and Giełwanowska (1987); Bednara and Rodkiewicz (1985); Bednara et al. (1986, 1995); Rodkiewicz et al. (1986, 1992)
		*Clarkia elegans* and *Lysimachia thyrsiflora*	Rodkiewicz et al. (1986)
		*Stangeria eriopus*	Rodkiewicz et al. (1986, 1988a, 1992); Rodkiewicz and Duda (1988)
		*Impatiens sultani* and *Lonicera japonica*	Brown and Lemmon (1988b)
		*Cypripedium californicum*	Brown and Lemmon (1996, 1998)
C	Neutral equatorial during telophase	*Chamaedorea Karwinskiana* *Tropaeolum peregrinum* *Ribes rubrum* *Podocarpus macrophylla* *Paeonia tenuifolia,* and *Campanula rapanculoides* *Pteridium aquilinum* *Dryopteris borreri* *Lycopersicon peruvianum* *Datura inoxa* and *Nicotiana tabacum* and *Antirrhinum majus* *Polystichum loncitis* *Solanum nigrum* Orchids “Sabine Queen” *Lilium longiflorum* *Ophioglossum petiolatum* *Psilotum nudum* *Ginkgo biloba* *Taranna gracilipes* *Armoracia rusticana* *Arabidopsis thaliana* *Larix europea* Orchids “Vista Rainbow” *Tinantia erecta*	Suessenguth (1921) Sugiura (1928) Geneves (1967, 1971) Vasil and Aldrich (1970) Dietrich (1973) Sheffield and Bell (1979) Sheffield and Bell (1979); Sheffield et al. (1983) Pacini and Juniper (1984) Dupuis et al. (1988) Bednara and Rodkiewicz (1988) Bhandari and Sharma (1988) Brown and Lemmon (1991b) Dickinson and Heslop-Harrison (1970); Tanaka (1991) Brown and Lemmon (2001a, b) Lee (1982); Gabarayeva (1985); Tchórzewska et al. (1996, 2011) Wolniak (1976); Wang et al. (1988); Brown and Lemmon (2005) Vinckier and Smets (2007) Winiarczyk et al. (2007) Brownfield et al. (2015) Rodkiewicz et al. (1984a, b); Bednara and Rodkiewicz (1988) Brown and Lemmon (1991b) personal communication
C	Lateral equatorial during telophase	*Marsilia quadrifolia* *Delphinium elatum* *Nymphaea alba* *Ophioglossum vulgatum*	Marquette (1908) Bednara et al. (1995) Rodkiewicz and Duda (1988); Rodkiewicz et al. (1988a, 1989, 1992); Bednara et al. (1995) Giełwanowska et al. 2003
M	Capsular	*Larix decidua* *Lavatera* ***Larix europea*** *Thespis* *Larix dahurica* *Cucumis sativus* *Gladiolus gandaviensis* and *G. primulinus* *Malva sylvestris* *Lavatera trimestris* *Lavatera thuringiaca* *Gossypium arboreum* and *Alcea rosea*	Nĕmec (1930) Byxbee (1900) Devise (1922) Youngman (1927) Prosina (1928) Heimlich (1929) Migdalska (1934) Rodkiweicz and Duda (1988); Rodkiewicz et al. (1988a); Kudlicka and Rodkiewicz (1990) Kudlicka and Rodkiewicz (1990) Tchórzewska et al. (2008, 2013) Denham (1924); Tchórzewska et al. (2013)
C	Capsular equatorial during telophase	*Hemerocallis fulva* fl. p*leno* ***Equisetum palustre*** ***Equisetum limosum*** *Helleborus foetidus* ***Ginkgo biloba*** *Nephrodium molle* *Chondrilla juncea*	Fullmer (1899); Sienicka (1929) Lewitsky (1926) Jungers (1934) Nicolosi-Roncati (1910); Echlin and Godwin (1968) Mann (1924) Senjaninova (1927) Kościńska-Pająk and Bednara (2003)
I	Lateral capsular equatorial during telophase	*Petunia violacea* ***Nymphaea alba***	Matsuda (1928) Guignard (1898)
M	Neutral	*Tetraclinis articulata* ***Equisetum variegatum*** *Allium sativum* *Allium ampeloprasum*	Saxtona (1913) Lenoir (1934) Winiarczyk (2009) personal communication
I	Neutral-polar	*Lupinus albus* *Equisetum limosum*	Milovidov (1928) Becker and Siemaszko (1936)
M	Polar	*Limodorum abortivum*	Gugnarda (1898)
I	Capsular-polar	*Magnolia Yulan* *Riccia Frostii*	Gugnarda (1898) Black (1913)
	Monoplastidic	*Rhynchostegium serrulatum* *Amblystegium riparium* *Atrichum undulatum* and *Entodon seductrix* *Marattia* *Angiopteris evecta* *Phaeoceros laevis* and *Notothylas breutelii* *Isoetes melanopoda* *Selaginella arenicola*	Brown and Lemmon (1982a, 1982b) Brown and Lemmon (1982c) Brown and Lemmon (1987a, 1987b), Brown and Lemmon (1997) Brown and Lemmon (2001a) Brown and Lemmon (1990) Brown and Lemmon (1987a, 1987b, 1991a) Brown and Lemmon (1985, 1991a)

In bold—species with chondriokinesis assigned in recent studies to different types than those suggested by Bąkowski (1938)

*M* main, *C* complex, *I* intermediate chondriokinesis types (acc. to classification proposed by Bąkowski 1938)

### *Author contribution statement*

DT–conceived the study, interpreted the data, wrote the manuscript.
